# Biomimetic Remineralization of Demineralized Dentine Using Scaffold of CMC/ACP Nanocomplexes in an In Vitro Tooth Model of Deep Caries

**DOI:** 10.1371/journal.pone.0116553

**Published:** 2015-01-14

**Authors:** Zhen Chen, Shansong Cao, Haorong Wang, Yanqiu Li, Anil Kishen, Xuliang Deng, Xiaoping Yang, Yinghui Wang, Changhong Cong, Huajun Wang, Xu Zhang

**Affiliations:** 1 School of Stomatology, Hospital of Stomatology, Tianjin Medical University, Tianjin, PR China; 2 Discipline of Endodontics, Faculty of Dentistry, University of Toronto, Toronto, Canada; 3 Department of Geriatric Dentistry, Peking University School and Hospital of Stomatology, Beijing, PR China; 4 The Key Laboratory of Beijing City on Preparation and Processing of Novel Polymer, Beijing University of Chemical Technology, Beijing, PR China; 5 School of Energy and Environment Engineering, Hebei University of Technology, Tianjin, PR China; Texas A&M University Baylor College of Dentistry, UNITED STATES

## Abstract

Currently, it is still a tough task for dentists to remineralize dentine in deep caries. The aim of this study was to remineralize demineralized dentine in a tooth model of deep caries using nanocomplexes of carboxymethyl chitosan/amorphous calcium phosphate (CMC/ACP) based on mimicking the stabilizing effect of dentine matrix protein 1 (DMP1) on ACP in the biomineralization of dentine. The experimental results indicate that CMC can stabilize ACP to form nanocomplexes of CMC/ACP, which is able to be processed into scaffolds by lyophilization. In the single-layer collagen model, ACP nanoparticles are released from scaffolds of CMC/ACP nanocomplexes dissolved and then infiltrate into collagen fibrils via the gap zones (40 nm) to accomplish intrafibrillar mineralization of collagen. With this method, the completely demineralized dentine was partially remineralized in the tooth mode. This is a bottom-up remineralizing strategy based on non-classical crystallization theory. Since nanocomplexes of CMC/ACP show a promising effect of remineralization on demineralized dentine via biomimetic strategy, thereby preserving dentinal tissue to the maximum extent possible, it would be a potential indirect pulp capping (IPC) material for the management of deep caries during vital pulp therapy based on the concept of minimally invasive dentistry (MID).

## Introduction

Dental caries is a disease caused by the by-products (organic acids) of bacteria in the biofilm on tooth enamel or dentine; the organic acids produced by bacteria damage dental hard tissues and structures, leading to tooth decay or cavities. Despite the advancement in the dental materials and instruments of preventive dentistry over the past few decades, it is a major problem in dentistry affecting individuals of all races, ages, culture and socioeconomic background [1]. The early enamel caries can heal or be remineralized through improving the oral hygiene and remineralization treatments, but if lesions continually develop in the enamel or even in the dentine under the enamel, resulting in deep caries, the infected tooth tissues should be removed and then restorative treatment is carried out. However, excess removal of tooth tissues tends to make carious teeth fracture more easily and may cause accidental pulp exposure. It is recognized that carious dentin actually consists of two distinct layers with different ultramicroscopic and chemical structures [2]. The outer layer (infected dentine) is irreversibly denatured, infected and incapable of being remineralized, and this layer should be removed; the inner layer (affected dentine) is reversibly denatured, not infected, and capable of being remineralized and thus it should be preserved [2, 3]. Therefore, the remineralizion of non-infected carious dentine is significant for retaining tooth tissues to a maximum degree during vital pulp therapy, which is in line with the concept of minimally invasive dentistry (MID) [4].

Currently, it is still a challenge to remineralize remaining demineralized dentine in deep caries. Dentine remineralization is more difficult than enamel remineralization due to the abundant presence of organic matrix in dentine. This could be attributed to an accepted notion that dentine remineralization occurs neither by spontaneous precipitation nor by nucleation of mineral on the organic matrix (mainly type I collagen), but by growth of residual crystals in the lesions [5, 6]. It is possible to remineralize lesions extending into the dentine with fluoride [7, 8]; however, this process depends on the amount of residual crystals and supplement of calcium and phosphate ions [9], taking a considerably long time, which is unacceptable in clinical practice. Calcium hydroxide (Ca(OH)_2_) as an IPC material, shows remineralizing effect on demineralized dentine *in vitro* in presence of remineralizing buffer [10]. It can also remineralize remaining carious dentine *in vivo* according to radiographic and histological results [11, 12]; however, the remineralizing rationale of calcium hydroxide and its effect on mineralization of collagen have not been elucidated exactly [10]. In fact, collagen matrix in the affected dentine provides a scaffold to be remineralized and thus remineralizing this scaffold could enhance the remineralization of deep dentine caries [13]. Hopefully, with the advancement of understanding on biomineralization of dental hard tissues, a biomimetic strategy and methodology could be developed for remineralization of demineralized collagen in carious dentine.

DMP1, a member of the small integrin binding ligand N-linked glycoprotein family, is identified as acidic noncollagenous protein expressed during the initial stages of mineralized matrix formation in bone and dentine [14], which is critical for regulation of biomineralization of bone and dentine. This protein contains a large number of acidic domains and multiple phosphorylation sites [14]. It is proposed that amorphous calcium phosphate (ACP) nanoclusters can be stabilized by self-assembled DMP1 in solution to form nanocomplexes, showing specific affinity toward the gap zone of collagen [15]. Thus, these nanocomplexes of DMP1/ACP as a precursor to apatite can enter the gap zone of collagen and finally transform into hydroxyapatite (HAP) crystals to mineralize collagen. Inspired from this *in vitro* behavior of DMP1 in the process of collagen mineralization, some analogues of DMP1, such as polyacrylic acid (PAA) and polyaspartic acid (PASA) have been used to stabilize ACP to mineralize collagen in demineteralized dentine *in vitro* [16–19]. It should be noted that the mineralization types of the collagen in dentine can be divided into the intrafibrillar and extrafibrillar mineralization [20–22]. Intrafibrillar mineralization forms by minerals depositing within the gap zones of collagen fibril and extending along the microfibrillar spaces within the fibril, while extrafibrillar mineralization occurs within the interstitial spaces separating the collagen fibrils [20–22]. The former is identified to contribute majorly to the mechanical properties of dentine [16, 23, 24]. Therefore, biomimetic strategy is hopeful to accomplish intrafibrillar mineralization of collagen and thereby remineralize demineralized dentine to the greatest extent possible.

Finding analogues of acidic non-collageneous proteins that are capable of stabilizing ACP is an important strategy for the development of novel biomimetic-remineralizing agents. So far, PASP and PAA have been proved to be able to chelate calcium ions due to its rich carboxyl groups and thus stabilize ACP in solution to form amorphous nanoprecursors. It was reported that carboxymethyl chitosan (CMC), the derivative of chitosan with rich carboxyl groups, can retard or inhibit the rate of spontaneous calcium phosphate precipitate [25]. Thus, water soluble CMC has a potential to facilitate formation of ACP nanoprecursors in solution by its chelating capacity. However, up to now, the formation of nanocomplexes of CMC/ACP in solution has not been characterized. CMC is biodegradable, biocompatible, nontoxic and antibacterial [26]; compared with PAA and PASA, it can be processed into scaffolds by lyophilization as indirect pulp capping agents conveniently.

Most of *in vitro* studies on remineralization of dentine were carried out in bulk solution [5–7, 9, 16, 18]. In this study, a tooth model of deep caries combined with the circulation of simulated body fluid (SBF), simulating the real clinical environment of deep caries during vital pulp therapy as far as possible, was used to test the remineralizing effect of CMC/ACP scaffolds. The hypothesis of this study was that nanoparticles of ACP from CMC/ACP scaffolds gradually dissolved with SBF can accomplish intrafibrillar remineralization of demineralized collagen, thereby facilitating remineralization of dentine.

## Materials and Methods

### 2.1 Synthesis of CMC and preparation of CMC/ACP scaffolds

CMC was synthesized according to ref.[27]. First, 13.5 g NaOH was dissolved in 100 ml of solvent comprising 20 ml water and 80 ml 2-propanol (Sigma-Aldrich Inc., St. Louis, MO, USA), to which 10 g chitosan (85% deacetylated, Sangon Co., Ltd. (Shanghai, China) was gradually added under stirring (1000 rpm). This mixture was allowed to swell and alkalize at 25°C for 2 h. Next, 15 g monochloroacetic acid (Sigma-Aldrich Inc., St. Louis, MO, USA) dissolved in 20 ml 2-propanol was added dropwise to the mixture in 30 min. The activated chitosan was reacted with the acid for 4 h at 50°C, which was stopped by addition of 200 ml of 70% ethanol. After that, the resulting precipitate was desalted by dialysis and then dried under reduced pressure at 25°C to obtain CMC powder.

CMC gel was first prepared by mixing 2.5 g CMC into 40ml water under stirring (1000 rpm) until the CMC powder was completely dissolved. Then, 0.498 g K_2_HPO_4_ was added into the CMC gel under stirring (500 rpm). Next, 0.555 g CaCl_2_ was added into 10 ml deionized water and this solution was added dropwise into the CMC gel under stirring for 5 min to form CMC/ACP gel. The final concentrations of calcium and phosphate ions were 100 mM and 60 mM, respectively. This gel was immediately frozen at -80°C for 2 h and then lyophilized in vacuum freeze dryer (Lgj-B10, Beijing, China) for 6 h to form CMC/ACP scaffolds.

### 2.2 Mineralization of collagen fibrils using a single-layer collagen model

Collagen fibrils were used to test the mineralizing ability of CMC/ACP scaffolds dissolved by SBF. The preparation of assembly of collagen fibrils on TEM grids as a single-layer collagen model referred to ref. [17]. Briefly, 0.5 g of the lyophilized type I collagen powder derived from calf skin (Sigma-Aldrich) was first mixed overnight with 5 ml of aqueous acetic acid (50 mM, pH 2.5), which was centrifuged at 4000 rpm for 15 min and then the supernatant was collected and stored at 4°C. Fifteen μl of collagen solution was dropped onto a TEM grid of gold on filter paper to remove excess of water; the grids were transferred onto a new piece of filter paper and dropped with 15 μl of Hepes buffer (10 mM, pH 7.4) containing NaCl (150 mM) for 30 min to trigger collagen assembly into fibrils and its subsequent precipitation. One g of CMC/ACP scaffold obtained in section 2.1 was put into 10 ml of SBF solution, over which the collagen-coated grids were floated upside-down for 48 h as a treatment group (n = 6). In a control group (n = 6), the grids were floated over SBF solution.

### 2.3 Preparation of the tooth model of deep caries

**Figure 1:**
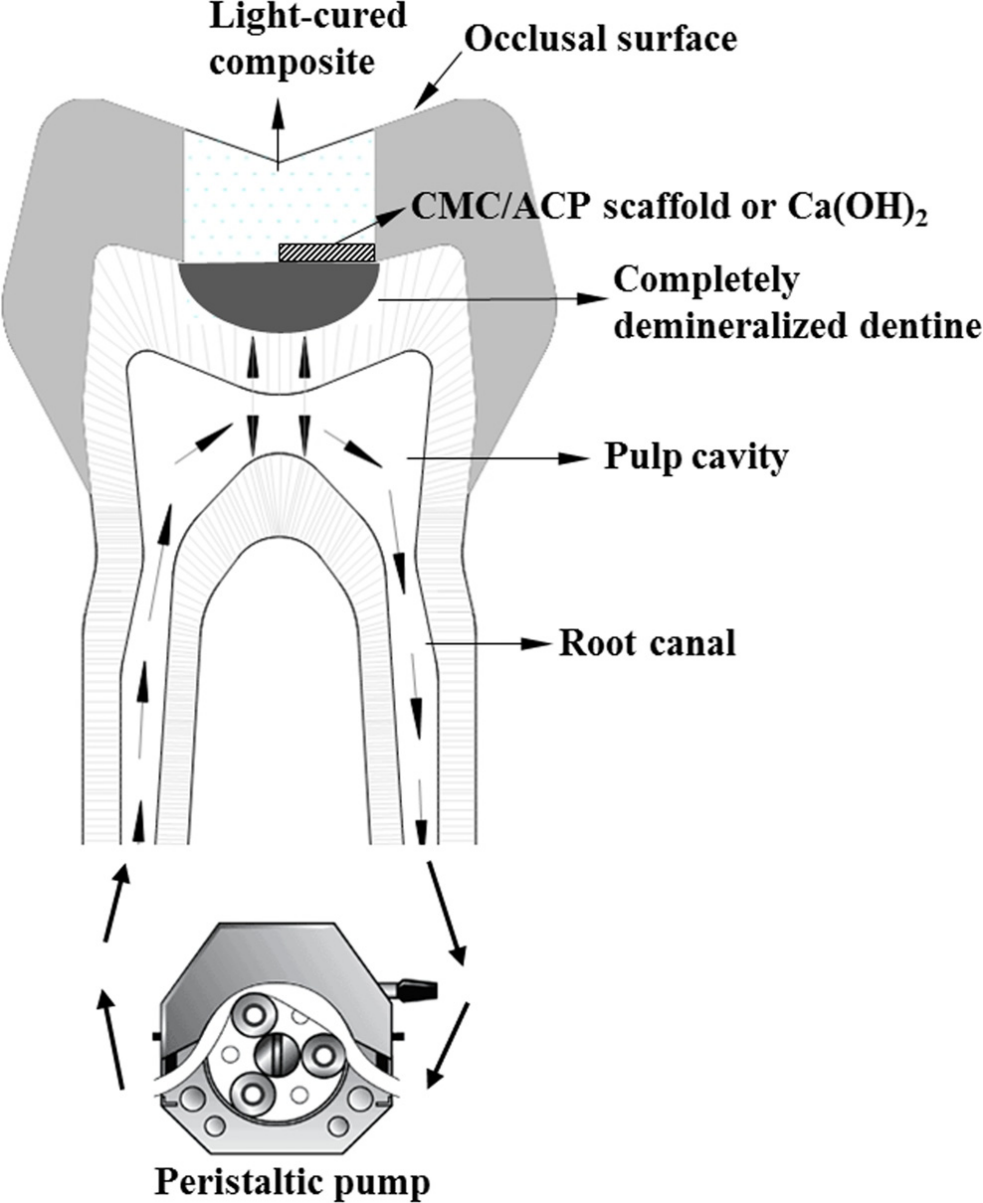
The scheme of tooth model of deep caries.

The institutional ethics committee of the Tianjin Medical University approved the collection and use of extracted human teeth for the experiments conducted in this study (TMUhMEC2012019). Before we collected the participants’ teeth, the written informed consent was shown to the participants. With the signed informed consent, the collection of teeth was carried out; this procedure was approved by the ethics committees. Extracted non-carious human third molars (n = 20) from patients (n = 12) aged 20–35 years without visible evidence of cracks, maintained in phosphate buffered saline (PBS) were used in this study. Fig. 1 shows the schematic diagram of the tooth model of deep caries. For each tooth model, a cavity (ca.3mm×3mm×3mm) was prepared on the occlusal surface (Fig. 1). The average remaining dentine thickness was about 1.89±0.23 mm measured by Micro-CT (Skyscan1174, Kontich, Belgium) and calculated using affiliated CT-Analyser software. For better fluid transportation, about 2 mm of the apical part of the roots were removed by a slow-speed contra-angle handpiece (NSK, Japan). Pulp tissue in the pulp chamber and root canal was removed with barbed broachs followed by H_2_O_2_ irrigation. The root canals were prepared using K files (Dentsply International. Inc, DE, USA) with lubrication of EDTA (ethylenediaminetetracetic acid). The diffusing effectiveness was checked by putting a cotton pellets into the cavity followed by circulating water between root canals; the wetting of the pellets indicated successful diffusion. The outer surface of the tooth models and four lateral surfaces of the cavity were sealed with nail varnish; only the bottom of the cavity was exposed to 50 ml solution of EDTA (17%, w/w%), which was carried out for 1 week in a shaking incubator at 37°C to completely demineralize the bottom dentine. Followed by rinsing with deionized water for 30 min, the tooth model was immersed in 20 ml of 1 M NaCl (pH 7.0) at 25°C for 8 h. The treatments of 1 M NaCl can remove a major part of soluble and immobilized noncollagenous proteins (NCPs) in dentine [28]. With 30 min rinse, the sample was subsequently kept in sterile deionized (DI) water. To guarantee sufficient demineralization, a micro-CT examination was used to monitor the demineralizing degree of the samples.

### 2.4 Remineralization of the teeth model

Totally, twenty samples of the teeth model were used in this study; they were randomly assigned into CMC/ACP and Ca(OH)_2_ (Dycal^®^, Dentsply International. Inc, DE, USA.) groups (n = 10). In each group, all samples were first characterized using Micro-CT and then used for other characterizations. As shown in Fig. 1, one half of cavity bottom of a sample was covered with a CMC/ACP scaffold or with Ca(OH)_2_, and other half was sealed with light-cured composite (TransbondXT 3M USA). Then, all the cavities of the samples were densely sealed with the same composite. During the process of remineralization, SBF at 37°C was circulated at 1 ml/min through root canals and pulp cavity from a fluid reservoir containing 1 L of SBF, using plastic tubing connected to peristaltic pump (Fig. 1). The remineralizing period was two weeks. SBF with ionic composition similar with the human plasma was prepared according to ref. [19].

### 2.5 Characterization

**2.5.1 Characterization of CMC/ACP.** The size and morphology of the nano-complexes of CMC/ACP in the scaffolds was characterized using a TEM (JEM-1230, JEOL, Tokyo, Japan) at 110 kV. SAED was used to identify the nature and crystallinity of minerals. Samples for inspection by TEM were prepared by slowly evaporating a drop of the gel of CMC/ACP at room temperature on a 400 mesh copper grid, which was covered by a carbon support film.

The FTIR spectra of chitosan, the CMC, nano-complexes of CMC/ACP and synthesized ACP (synthesized by referring Ref. [29]) samples were recorded in transmission mode using the KBr pellet method. Spectra were collected in the range from 400 to 4000 cm^-1^ at 16 cm^-1^ resolution, with 100-time scans using an infrared spectrophotometer (Shimadzu 8400S, Tokyo, Japan).

To characterize the surface morphology of CMC/ACP scaffolds, the samples were first sputter-coated with platinum and then observed by a FE-SEM (JSM-6701F, JEOL, Japan) with a beam voltage at 5.0 kV.

To determine whether the scaffolds of CMC/ACP were still amorphous after lyophilization, the scaffolds were ground into powder for XRD characterization. XRD measurements were carried out using XRD-6000 X-ray diffractometer (Shimadzu, Tokyo, Japan) with Cu K_α_ radiation. The data were collected in the 2*θ* range of 10–50° at a scan rate of 2° per min.

**2.5.2 Characterization of mineralization of collagen fibrils using TEM.** After mineralization, grids were washed with MilliQ water for 10 minutes and subsequently put on filter paper to remove excess of water. Unstained grids were examined using transmission electron microscopy (JEM-1230, JEOL).

**2.5.3 Characterization of the remineralization of tooth model.** Before and after demineralization, all samples of tooth model were scanned by SkyScan 1174 compact X-ray micro-CT scanner (Micro Photonics, Allentown, PA, USA). The samples were scanned with a spatial resolution of 6.55 μm and their projection images were collected at 50 kV and 800 μA using 360° rotation with 0.7° per projection step. Aluminum filter with thickness of 1 mm was placed in the beam path to remove low-energy radiation, compared with the fluoride treatment. Two-dimension images of the samples in the sagittal plane were reconstructed using the CT-Analyser software (Skyscan1174, Kontich, Belgium). To obtain a stacked 2-D image, 200 sagittal virtual serial slices of a sample were selected to subject to maximum intensity projection (MIP) with the same software.

The stacked images were analyzed with Image J software (NIH, Bethesda, MD, USA) to produce an overall mineral profile within a standardized volume of interest (VOI). A rectangular box (500×900 μm^2^, width×depth) was plotted on a representative zone of the stacked image from approximately 100 μm on the outside of the lesion surface (air) to the sound region. Thus, the VOI was 0.59 mm^3^ [i.e. 500×900×6.55×200 μm^3^]. The effective remineralization depth (μm) was determined from the lesion surface to the position where the relative mineral content was 50%. Mineral loss (ΔZ, vol%μm) was calculated from the MD profiles of the samples by subtracting the area under the curve of completely demineralised dentine or remineralized dentine from the area under the curve of sound dentine within the effective remineralization depth. The rate of remineralization of samples was calculated according to the formulation: R = (ΔZ_d_-ΔZ_r_)/ΔZ_d_ (d, baseline data without remineralization treatment; r, data after remineralization treatment).

After the Micro-CT characterization, five samples were taken for field emission gun scanning electron microscope (FE-SEM) characterization. The dentine blocks in the bottom of teeth model were obtained by cutting enamel and sound dentine. The samples were first washed using DI water, then dehydrated using ethanol (at gradient concentration of 50%, 70%, 80% and 90% for 20 min and 100% for 2 h) and finally sputter-coated with platinum before FE-SEM and EDX characterization. The surface morphology and elemental compositions of the samples was characterized by a FE-SEM (JSM-6701F, JEOL, Japan) with a beam voltage at 5.0 kV. A quantitative elemental analysis of Ca and P was carried out by EDX analysis.

The other five samples were taken for TEM characterization. The dentine blocks removed from the teeth model were sectioned perpendicularly to a cornal-pulpal direction into 1 mm thick slices. After that, these slices were fixed in Karnovsky’s fixative and post-fixed in 1% osmium tetroxide. Then, these samples were dehydrated in an ascending ethanol series (50–100%), immersed in propylene oxide and embedded in epoxy resin. Ninety nm thick sections of completely demineralized and remineralized dentine were prepared and examined without further staining using TEM (JEM-1230, JEOL, Tokyo, Japan) at 80 kV.

### 2.6 Statistical analysis

The statistical analysis of remineralization depth, remineralization rate and Ca and P contents were carried out using pair-wise *T*-tests. The results are indicated as mean ± standard deviation. All statistical analysis was performed at a 95% level of confidence with the Statistical Package for Medical Science (SPSS ver. 10 for Windows, SPSS Inc., Chicago, IL, USA).

## Results

### 3.1 Characterization of CMC/ACP

**Figure 2:**
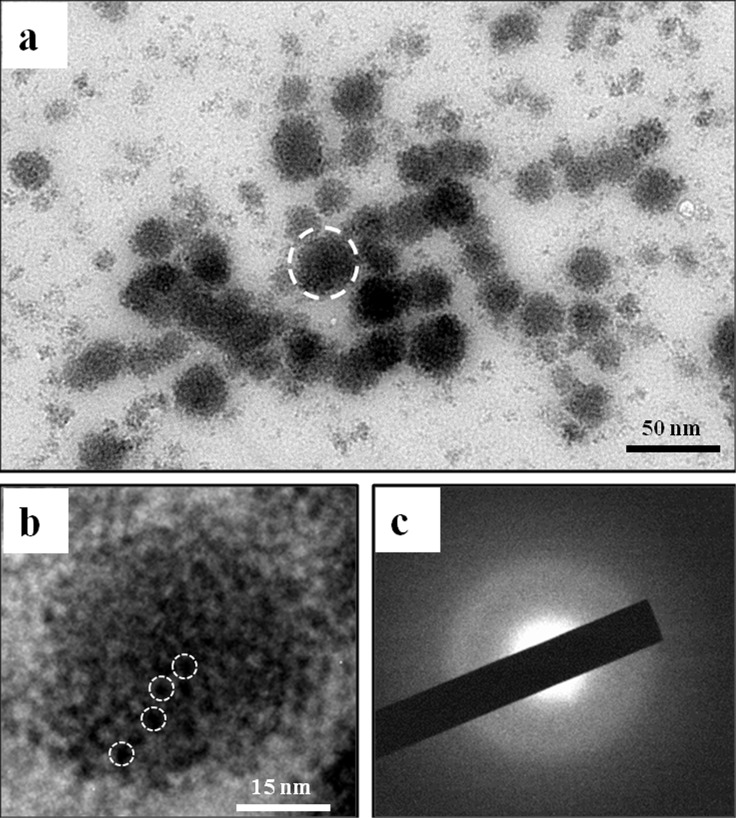
TEM and SAED characterization of nano-complexes of CMC/ACP. (a) Image of nanoparticles of ACP formed in presence of CMC (nanocomplexes of CMC/ACP). (b) Higher magnification of the typical nanoparticle indicated by a circle of white dash line in (a). This nanoparticle was composed of much smaller prenucleation clusters indicated by white dash line circles. (c) SAED of nanoparticles of CPP/ACP did not show obvious dot or ring pattern characteristic of crystal structure, which indicates that its main composition is amorphous phase.

TEM images of CMC/ACP gel did not show any crystallites and only the aggregates of nanoparticles with diameters of less than 50 nm were observed (Fig. 2(a)). Fig. 2(b) shows a typical aggregate composed of many smaller nanoparticles; these aggregates could be defined as nano-complexes of CMC/ACP. The image of SAED of the nanoparticles did not show obvious dot or ring pattern characteristic of crystal structure, which indicates that its main composition was amorphous phase (Fig. 2(c)).

**Figure 3:**
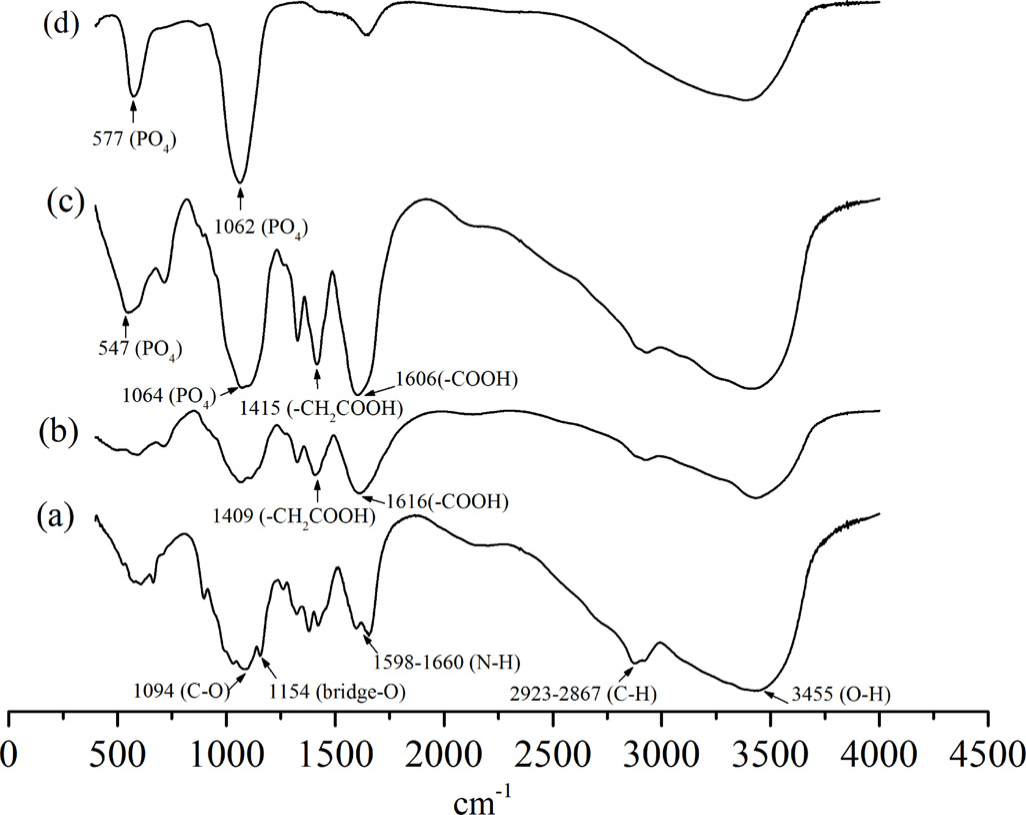
FTIR spectrum of (a), chitosan; (b), carboxymethyl chitosan; (c), nanocomplex of CMC/ACP; (d), synthesized ACP.

The basic characteristic peaks of chitosan in IR spectrum are shown in Fig. 3(a). Compared with the peaks of chitosan, the appearance of peaks at 1616 and 1409 cm^-1^ corresponding to the carboxy group (which overlaps with N-H bend) and -CH_2_COOH group (Fig. 3(b)), respectively, indicates the carboxymethylation on both the amino and hydroxyl groups of chitosan [26]. By contrast, in the IR spectrum of CMC/ACP (Fig. 3(c)), these two peaks shifted to 1606 and 1415 cm^-1^, respectively, indicating the coordination interaction between calcium ions and CMC [30]. The intense peaks located at 1062 and 577 cm^-1^ are assigned to PO_4_^3-^ of ACP (Fig. 3(d)), which could be identified in the spectrum of CMC/ACP despite of the peak shift (Fig. 3(c)).

**Figure 4:**
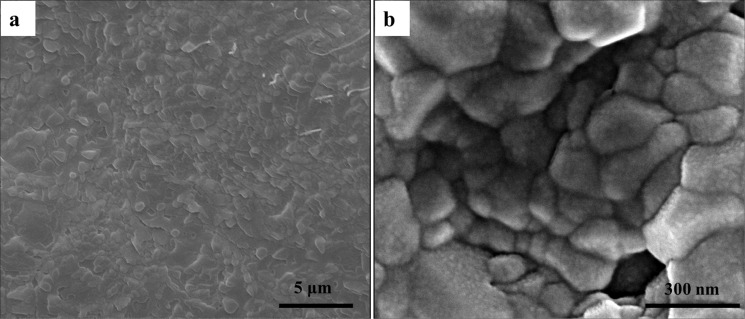
The surface morphology of scaffolds of CMC/ACP nanocomplexes characterized by SEM. (a), low magnification image showing irregular granuleson the sample surface; (b), high magnification image showing aggregation of nanoparticles on the granules.

Fig. 4 shows the surface morphology of CMC/ACP scaffolds. The irregular granules were found on the sample surface (Fig. 4(a)); at higher magnification, Fig. 4(b) shows the aggregation of nanoparticles on these granules.

**Figure 5:**
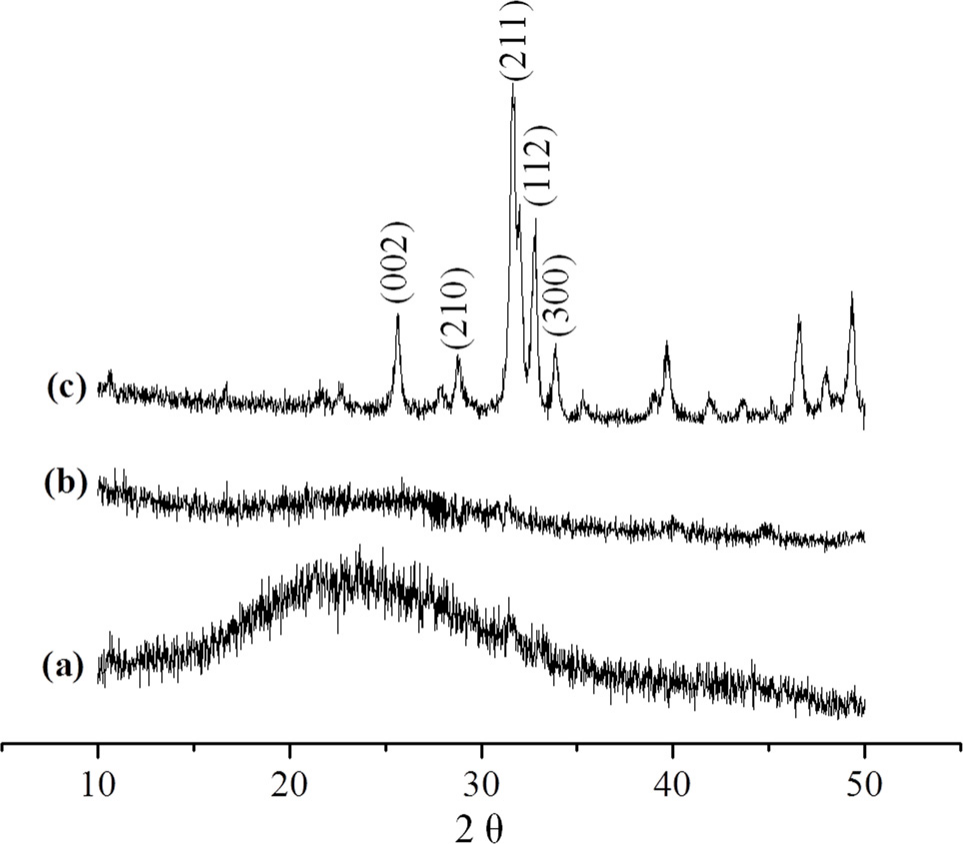
XRD spectrum of (a), CMC; (b), CMC/ACP; (c) HAP powder.

XRD was used to identify the mineral phase in the CMC/ACP scaffolds processed by lyophilization. In Fig. 5(a), since XRD of chitosan shows a sharp peak at 20°, the broad peak at 20° indicates the incorporation of other functional groups into chitosan, which decreases the crystalline of chitosan [31]. Compared with the spectrum of HAP powder, the spectrum of CMC/ACP lacked characteristic diffraction pattern of crystalline HAP (Fig. 5(c)), indicating an amorphous calcium phosphate phase associated with CMC (Fig. 5(b)).

### 3.2 Mineralization of collagen fibrils

**Figure 6:**
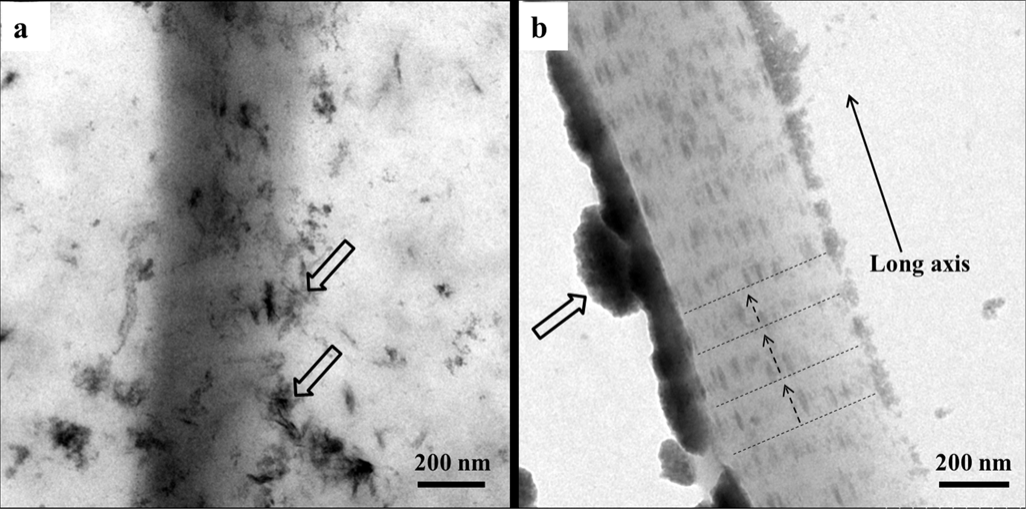
Representative TEM images of unstained samples of collagen fibrils. (a), a collagen fibril mineralized with SBF, the open arrows indicating needle-like crystals as extrafibrillar minerals; (b), a collagen fibril mineralized with nanocomplexes of CMC/ACP regained by dissolving their scaffolds in SBF solution, the open arrows indicating nanocomplexes of CMC/ACP, the dash lines indicating the cross-bandings formed by mineral crystals and the arrows of dash line denoting the orientation of mineral crystals.

Only need-like crystals were found around the collagen matrix after 48 h (Fig. 6(a)), which indicates that reconstituted type I collagen does not induce intrafibrillar mineralization in the absence of biomimetic analogues, even in SBF solution. In contrast, since the CMC/ACP scaffolds could be gradually dissolved in SBF solution to release nanocomplexes of CMC/ACP (Fig. 6(b)), ACP nanoparticles could be observed on the surface of collagen fibrils, which infiltrated into the collagen fibrils to induce mineralization. Also, apparent cross-bandings formed by mineral crystals, corresponding to the D-spacing of collagen molecules, could be observed within the fibrils (Fig. 6(b)), and the crystals arrayed along the long axis of collagen fibril, indicating intrafibrillar mineralization.

### 3.3 Characterization of remineralization of tooth model

**Figure 7:**
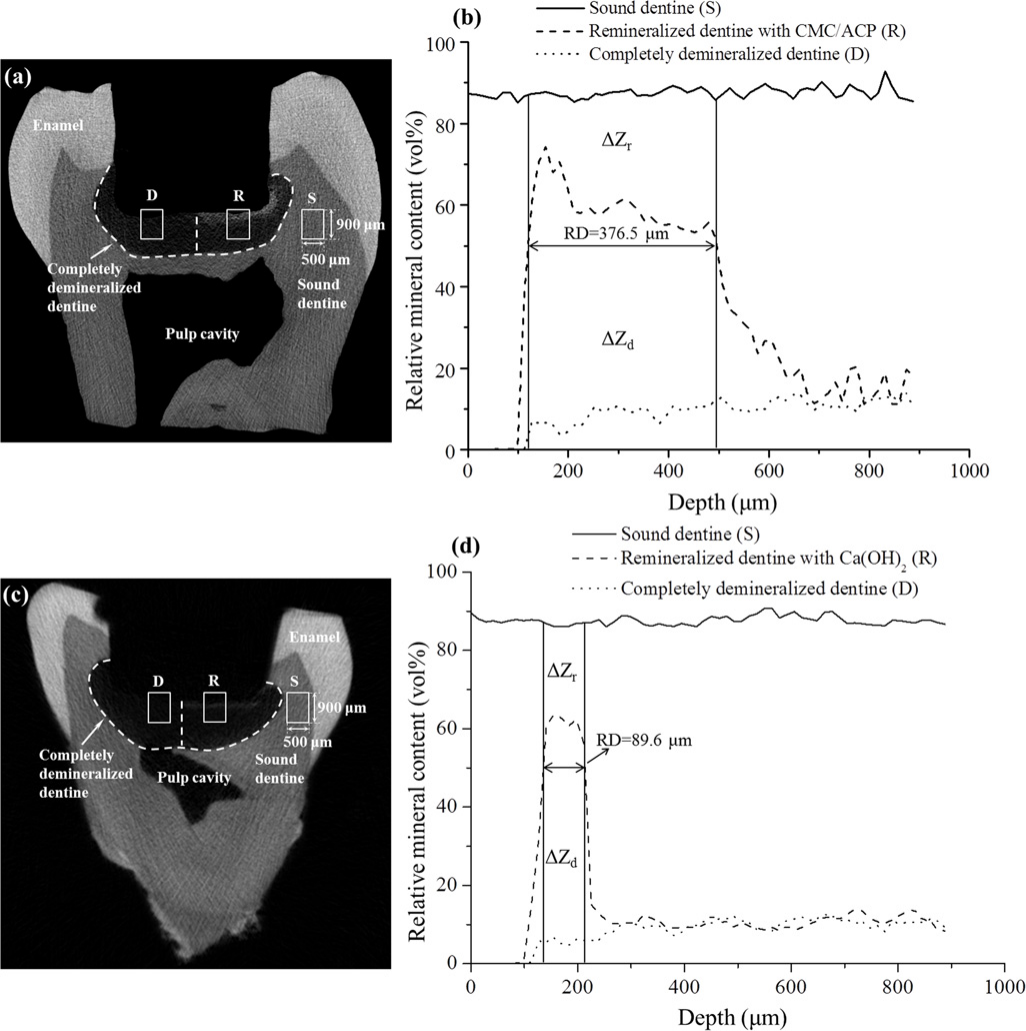
Representative Micro-CT images and mineral profiles of the samples of tooth model of deep caries. (a, b), CMC/ACP treatment group; (c, d), Ca(OH)_2_ treatment group.

**Figure 8:**
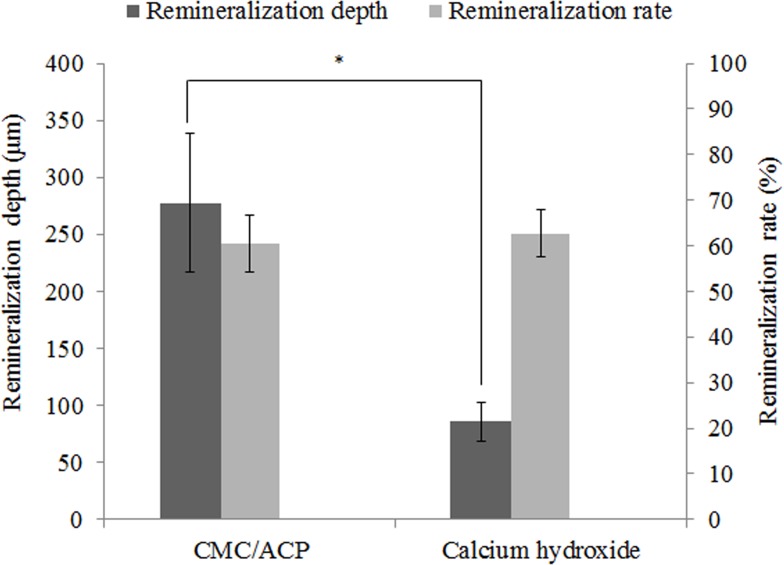
Results of remineralization depth and remineralization rate in CMC/ACP treatment group and Ca(OH)_2_ treatment group. The CMC/ACP treatment showed higher average value of effective remineralization depth compared with Ca(OH)_2_ treatment (*, P<0.05, n = 10), while the difference in remineralization rate between Ca(OH)_2_ and CMC/ACP groups was not statistically significant.

The typical micro-CT images and the corresponding mineral profiles show the remineralizing effect of CMC/ACP and Ca(OH)_2_ on the completely demineralized dentine in the bottom of the tooth model (Fig. 7). The area covered with CMC/ACP or Ca(OH)_2_ showed high CT intensity compared with the control area (Fig. 7(a,c)); however, the corresponding mineral profiles (Fig. 7(b,d)) of the representative samples shown in Fig. 7(a,c) demonstrate the different remineralizing effects. Ca(OH)_2_ treatment showed an abrupt decrease in mineral content, while CMC/ACP treatment resulted in a relatively gradual decrease. As shown in Fig. 8, no statistically significant difference in remineralization rate could be found between Ca(OH)_2_ and CMC/ACP groups, but CMC/ACP treatment led to a higher average value of effective remineralization depth compared with Ca(OH)_2_ treatment.

**Figure 9:**
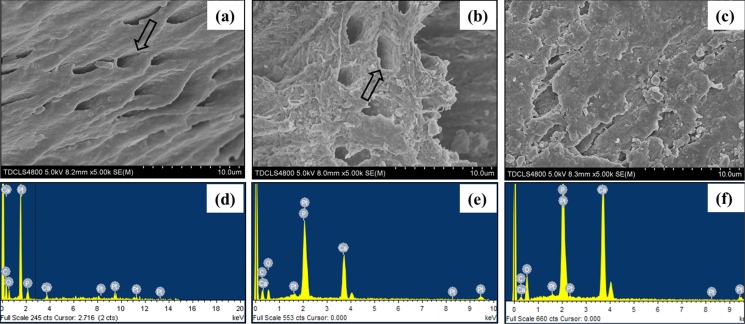
The surface morphology and elemental compositions of representative samples of completely demineralized dentine in the tooth model characterized by SEM and EDX. (a, d) the control sample, showing collapse orifices of dentinal tubule (open arrow); (b, e) the sample treated with CMC/ACP, showing renatured orifices of dentinal tubule (open arrow); (c, f) the sample treated with Ca(OH)_2_.

**Figure 10:**
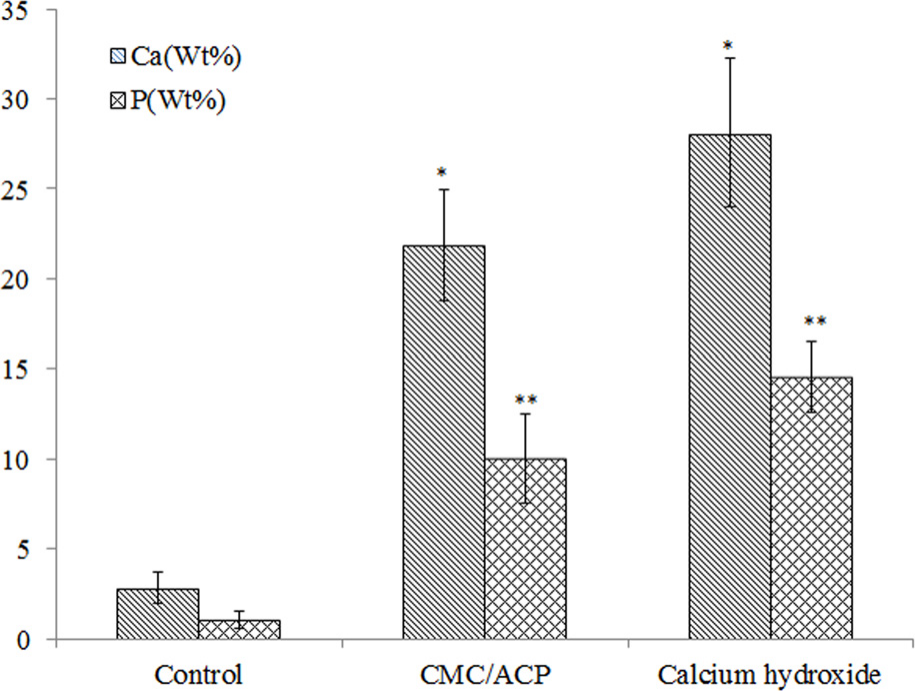
Calcium and phosphorus content in wt% of the surface of dentine samples with different treatments. (*, P<0.05, n = 5, compared to control; **, P<0.05, n = 5, compared to control)

In Fig. 9, the representative SEM images and the corresponding EDX results were shown of the sample treated with CMC/ACP and Ca(OH)_2_. Compared with the control parts of tooth model, CMC/ACP and Ca(OH)_2_ treatments significantly increased the content of calcium and phosphorus on the sample surfaces (Fig. 10). The surface of control dentine in the tooth model shows collapsed dentine collagen, on which the orifices of dentinal tubules shrank (Fig. 9(a)); almost no elements of calcium and phosphorus could be detected by EDX (Fig. 9(d)). By contrast, it was observed that the orifices of dentinal tubules reopened on the surface of dentine with CMC/ACP (Fig. 9(b)), indicating the ultrastructure of dentine collagen may partially restore; the EDX results showed evident calcium and phosphorus elements on the surface of dentine (Fig. 9(e)). Ca(OH)_2_ caused a significant mineral layer on the surface of dentine in the tooth model, covering the orifices of dentinal tubule (Fig. 9(c)); this layer also contained much calcium and phosphorus elements (Fig. 9(f)).

**Figure 11:**
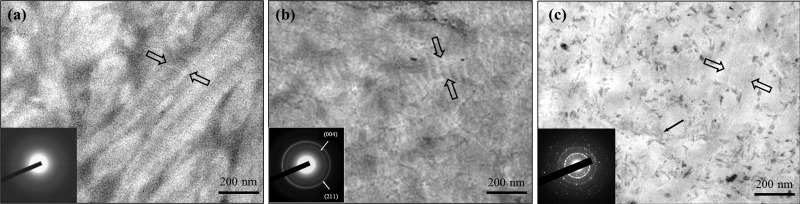
Representative TEM images of unstained samples of completely demineralized dentine in the tooth model and corresponding SAED patterns (inserts). (a) the control sample, showing faint banding characteristics (between open arrows) (b) the sample treated with CMC/ACP, showing characteristic type-I collagen D-bands (between open arrows), indicating intrafibrillar minerals within collagen fibrils after remineralization. (c) the sample treated with Ca(OH)_2_, showing faint banding characteristics (between open arrows) and needle-like crystals as extrafibrillar minerals.

The collagen fibrils in the control dentine in the tooth model exhibited only faint banding characteristics due to lack of intrafibrillar minerals (Fig. 11(a)); the corresponding SAED shows a diffuse pattern indicating the absence of minerals. By comparison, in the presence of CMC/ACP scaffold, characteristic D-bands of type-I collagen was observed in the remineralized zone and the corresponding SAED reveals characteristic discrete ring patterns of HAP, which indicates the intrafibrillar mineralization of collagen combined (Fig. 11(b)). For Ca(OH)_2_ treatment, many needle-like crystals were found in the sample, dispersing outside of collagen fibrils as exrafibrillar minerals; no obvious characteristic D-bands of collagen were identified (Fig. 11(c)), indicating that intrafibrillar mineralization is not a main remineralizing way of dentine collagen in the presence of Ca(OH)_2_.

## Discussion

The morphology of dentine was a honeycomb structure which consists of numerous dentinal tubules filled with dentinal fluid. The composition of dentinal fluid is similar to blood plasma [32]. This flowing fluid allows dentine to be permeable and hydrated, which maintains a substance exchange between dentine and pulp chamber. In this study, a tooth model of deep caries combined with circulation of SBF was designed to obtain a relatively real physiological environment compared to the conventional remineralization model (i.e. immersing samples into remineralizing medium). A similar tooth model has been used to assess the bioactivity of CMC scaffold combined with MTA (mineral trioxide aggregate) for dentine regeneration [33]. In our tooth model, the dentine was completely demineralized to eliminate the false positive results caused by the residual mineral crystals. The micro-CT results in this study demonstrate that our tooth model is able to mimic teeth with vital pulp and to be used to test the effects of IPC materials on remineralization of dentine in deep caries during vital pulp therapy. In this study, CMC/ACP scaffolds are temporarily filled for remineralizing treatments before permanent restoration; the requirement of the bonding between the scaffolds and the residual dentine will be further investigated in the future.

Amorphous calcium phosphate (ACP) is the initial solid phase precipitating from a highly supersaturated solution with respect to calcium phosphate, which is firstly described by Aaron S. Posner in the mid 1960s [34]. Owing to its excellent bioactivity, high cell adhesion, adjustable biodegradation rate and good osteoconduction, ACP has been widely applied in biomedical field, especially in orthopedic and dental fields [35, 36]. Since ACP can convert readily to stable crystalline phases such as octacalcium phosphate (OCP) or HAP, it is difficult to use ACP to remineralize dental hard tissues directly unless stabilized in some way [36]. In this study, TEM/SAED, FTIR, FE-SEM and XRD results indicate that CMC has a remarkable ability to stabilize nanoclusters of ACP into soluble nanocomplexes of CMC/ACP, thereby preventing them from growing to the critical size required for homogeneous nucleation, precipitation and phase transformation. The stable nanocomplexes of CMC/ACP in a minor diameter phase (<40 nm) can successfully infiltrate into collagen fibrils via the gap regions, but not be delivered as extrafibrillar particles. This could be attributed to the rich carboxyl groups in CMC that are able to chelate calcium ions, creating a charge to sequester counter ions (phosphate) [16]. The chain molecules of CMC bind to the spontaneously forming ACP nanoclusters and separate them individually (Fig. 2(b)), producing a metastable colloid of nanocomplexes of CMC/ACP. Although these nanoclusters can further assemble into nanoparticles, the growth of the nanoparticles in size and their transformation to HAP are inhibited by CMC. This biomimetic process was also known as “polymer-induced liquid-precursor”(PILP) [37, 38], which has been used to create nanoscale ACP to biomimetically mineralize type I collagen [18, 19]. It has been reported that this biomimetic process is independent of ion solubility products, and relatively insensitive to changes in pH and osmolarity, which is difficult to be explained by classical crystallization theory [39]. Accordingly, non-classical crystallization theory (pathway) was proposed to describe the biomimetic mineralization process based on PILP [40]. In the non-classical pathway, inorganic nanocrystals coated/stabilized with organic molecules can form larger mesocrystals via self-assembly and crystallographic alignment [39]. These mesocrystals work as intermediates for the formation of single macroscopic crystals. In our study, the stabilizing effect of CMC on ACP was similar to that of PAA or PASP, which indicates that it is an effective strategy for us to develop a novel biomimetic-remineralizing agent by seeking analogues of acidic non-collageneous proteins that are capable of stabilizing ACP.

In this study, nanocomplexes of CMC/ACP can be processed into scaffolds as a potential IPC material by lyophilization, the mineralizing effect of CMC/ACP on collagen was tested using the single-layer collagen model and the tooth model, respectively. In the experiment of former model, it was proved that CMC/ACP scaffolds can be dissolved to regain nanocomplexes of CMC/ACP in SBF solution and the ACP nanoparticles released can induce intrafibrillar mineralization of collagen. In the tooth model, a circulation of SBF with ionic composition representing human blood plasma was set up in this model to slowly dissolve CMC/ACP scaffolds for the release of ACP nanoparticles and provide a supersaturated environment with respect to HAP as well. In these two models, the ACP nanoparticles fitting the size of gap zones of collagen can enter the inside of collagen fibrils through the gap zones and then replace free and loosely bound water within collagen, which recapitulates the progressive dehydration mechanism of natural biomineralization [41]. The disordered ACP phase is a precursor to crystalline HAP and can finally transform into crystalline apatite mineral, thereby accomplishing intrafibrillar mineralization of collagen.

This manner of collagen mineralization, regarded as a bottom-up strategy based on non-classical crystallization theory, is different from the previous top-down strategy based on the classical crystallization theory [42]. In top-down approaches, phosphate or carboxyl groups are introduced onto collagen as nucleation sites by grafting peptides or polysaccharides containing these functional groups [43, 44] or by chemical phophorylation of collagen [45, 46]. With the top-down approaches, the mineralization of collagen carries out via ion-by-ion addition to the nucleation sites or pre-existing seed crystallites. In this study, the remineralization of dentine with calcium hydroxide could be attributed to the top-down manner in the tooth model. Here, calcium hydroxide provides a slowly releasing source of calcium and hydroxyl ions and maintain an alkaline environment, which facilitates the nucleation of the calcium and phosphate ions from SBF to form an initial ACP phase on the surface of collagen fibrils. In the absence of ACP-stabilizers, it is difficult to control the size of ACP and prevent it from transforming into apatite phase. These ACP precipitates on the surface of collagen and cannot enter inside of collagen fibrils and finally transform to mineral crystals as extrafibrillar mineralization, which was proved by the single-layer collagen model and the tooth model (Fig. 6(a) and Fig. 11(c)). Therefore, it is hard to induce intrafibrillar mineralization of collagen using top-down strategy as minerals will rapidly deposit on the surface of collagen fibrils, which hinders mineral ions from getting into the inside of collagen fibrils to nucleate. The above-mentioned discussion explains the phenomenon of the lack of intrafibrillar mineralization in Fig. 11(c), and indicates that Ca(OH)_2_ can induce extrafibrillar remineralization of collagen in completely demineralized dentine, but it is difficult for it to induce intrafibrillar remineralization of collagen *in vitro* in the absence of ACP-stabilizers. Conversely, the bottom-up strategy of collagen mineralization is independent of seed crystallites and ion transport. In the tooth model, as indicated by Micro-CT and TEM results (Fig. 7 and Fig. 11), SBF circulation itself did not induce significant remineralization of completely demineralized collagen in dentine, which further supports the ideal that CMC/ACP is based on the bottom-up strategy to mineralized collagen, independently of ion transport. In addition, some studies demonstrate that besides the analogues stabilizing ACP, template analogues of matrix phosphoproteins such as polyvinylphosphonic acid, sodium trimetaphosphate or sodium ascorbyl phosphate were needed for guiding intrafibrillar apatite deposition [18, 19, 42]. However, some more recent studies indicate that in absence of the template biomimetic analogues, type I collagen alone can acting as templates for electrostatic attraction of ACP nanoparticles and guide formation of intrafibrillar apatite within the gap zones to accomplish intrafibrillar mineralization of collagen [16, 17]. In this study, although the template biomimetic analogues were not employed, intrafibrillar mineralization of dentine collagen was accomplished by using nanocomplexes of CMC/ACP itself, which could be explained by the latter opinion mentioned above. Therefore, this study demonstrates that ACP nanoparticles from scaffolds of CMC/ACP nanocomplexes can accomplish intrafibrillar remineralization of demineralized collagen; this method also can mineralize dentine collagen, thus helping remineralization of demineralized dentine.

It should be noted that remineralization effect of IPC materials itself may not be sufficient for the management of deep caries. Antibacterial ability and induction activity for reparative dentinogenesis are also deeply concerned with the effect of treatments for deep caries. Antibacterial property of calcium hydroxide is primarily attributed to its highly alkaline pH of about 12.5 [47]. At such a high pH, the structure of enzymes is denatured, leading to the loss of biological activity of enzymes and the eventual cell death [48]. However, due to its limited solubility and diffusibility, calcium hydroxide may only kill the bacteria in superficial layers of demineralized dentine in deep caries [48]. In contrast, owing to the antibacterial property of CMC and good solubility of CMC/ACP scaffolds, the nanocomplexes of CMC/ACP may penetrate into deep of demineralized dentine to inhibit bacteria, which is implied by the different remineralization depths caused by Ca(OH)_2_ and CMC/ACP (Fig. 8). In addition, calcium hydroxide has been shown to enhance recruitment, migration, proliferation and mineralization of dental pulp stem cells, which facilitate reparative dentinogenesis [48]. It is summarized that calcium hydroxide causes reparative dentinogenesis by activating certain specific pathways in the pulp, such as modulation of expression of BMP2 (bone morphogenetic protein 2), BSP (bone sialoprotein) and osteopontin with its released ions [48].

In this study, although nanocomplexes of CMC/ACP shows a promising effect of remineralization on demineralized dentine, if CMC/ACP works as an IPC material, its antibacterial property and potential for induction of reparative dentinogenesis should be further investigated in our future study. Additionally, how to regulate and control transformation of ACP to HAP and enhance the mineralization depth of dentine should be investigated in future.

## Conclusions

In this study, CMC can stabilize ACP to form nanocomplexes of CMC/ACP, which is able to be processed into scaffolds by lyophilization. In the single-layer collagen model and tooth model of deep caries, ACP nanoparticles are released from scaffolds of CMC/ACP nanocomplexes to accomplish intrafibrillar mineralization of collagen using bottom-up strategy based on non-classical crystallization theory, thus facilitating remineralization of demineralized dentine. Since nanocomplexes of CMC/ACP show a promising effect of remineralization on demineralized dentine via biomimetic strategy, thereby preserving dentinal tissue to the maximum extent possible, it would be a potential IPC material for the management of deep caries based on the concept of MID.
